# Phase I dose-escalation and pharmacokinetic study of temozolomide (SCH 52365) for refractory or relapsing malignancies

**DOI:** 10.1038/sj.bjc.6690802

**Published:** 1999-11

**Authors:** M Brada, I Judson, P Beale, S Moore, P Reidenberg, P Statkevich, M Dugan, V Batra, D Cutler

**Affiliations:** The Royal Marsden NHS Trust and the Institute of Cancer Research, Downs Road, Sutton, Surrey, SM2 5PT, UK; 637 South Chestnut Street, Westfield, NJ 07090, USA; Schering-Plough Research Institute, 2000 Galloping Hill Road, Kenilworth, NJ 07033, USA

**Keywords:** oral, cytotoxic, chemotherapy, pharmacokinetics, dose escalation

## Abstract

Temozolomide, an oral cytotoxic agent with approximately 100% bioavailability after one administration, has demonstrated schedule-dependent clinical activity against highly resistant cancers. Thirty patients with minimal prior chemotherapy were enrolled in this phase I trial to characterize the drug's safety, pharmacokinetics and anti-tumour activity, as well as to assess how food affects oral bioavailability. To determine dose-limiting toxicities (DLT) and the maximum tolerated dose (MTD), temozolomide 100–250 mg m^−2^ was administered once daily for 5 days every 28 days. The DLT was thrombocytopenia, and the MTD was 200 mg m^−2^ day^−1^. Subsequently, patients received the MTD to study how food affects the oral bioavailability of temozolomide. When given orally once daily for 5 days, temozolomide was well tolerated and produced a non-cumulative, transient myelosuppression. The most common non-haematological toxicities were mild to moderate nausea and vomiting. Clinical activity was observed against several advanced cancers, including malignant glioma and metastatic melanoma. Temozolomide demonstrated linear and reproducible pharmacokinetics and was rapidly absorbed (mean *T*_max_ ~1 h) and eliminated (mean t_1/2_ = 1.8 h). Food produced a slight reduction (9%) in absorption of temozolomide. Temozolomide 200 mg m^−2^ day^−1^ for 5 days, every 28 days, is recommended for phase II studies. © 1999 Cancer Research Campaign


*British Journal of Cancer* (1999) **81**(6), 1022–1030
© 1999 Cancer Research Campaign
Article no. bjoc.1999.802

### Phase I dose-escalation and pharmacokinetic study of temozolomide (SCH 52365) for refractory or relapsing malignancies

**M Brada[1], I Judson[1], P Beale[1], S Moore[1], P Reidenberg[2], P Statkevich[3], M Dugan[3], V Batra[3] and D Cutler[3]**

[1]The Royal Marsden NHS Trust, and the Institute of Cancer Research, Downs Road, Sutton, Surrey, SM2 5PT, UK; [2]637 South Chestnut Street Westfield, NJ 07090, USA; [3]Schering-Plough Research Institute, 2000 Galloping Hill Road, Kenilworth, NJ 07033, USA

**Summary** Temozolomide, an oral cytotoxic agent with approximately 100% bioavailability after one administration, has demonstrated schedule-dependent clinical activity against highly resistant cancers. Thirty patients with minimal prior chemotherapy were enrolled in this phase I trial to characterize the drug's safety, pharmacokinetics and anti-tumour activity, as well as to assess how food affects oral bioavailability. To determine dose-limiting toxicities (DLT) and the maximum tolerated dose (MTD), temozolomide 100–250 mg $m^{-2}$ was administered once daily for 5 days every 28 days. The DLT was thrombocytopenia, and the MTD was 200 mg $m^{-2}$ $day^{-1}$. Subsequently, patients received the MTD to study how food affects the oral bioavailability of temozolomide. When given orally once daily for 5 days, temozolomide was well tolerated and produced a non-cumulative, transient myelosuppression. The most common non-haematological toxicities were mild to moderate nausea and vomiting. Clinical activity was observed against several advanced cancers, including malignant glioma and metastatic melanoma. Temozolomide demonstrated linear and reproducible pharmacokinetics and was rapidly absorbed (mean $T_{max}$ ~1 h) and eliminated (mean $t_{1/2}$ = 1.8 h). Food produced a slight reduction (9%) in absorption of temozolomide. Temozolomide 200 mg $m^{-2}$ $day^{-1}$ for 5 days, every 28 days, is recommended for phase II studies. © 1999 Cancer Research Campaign

**Key words**: oral; cytotoxic; chemotherapy; pharmacokinetics; dose escalation

Temozolomide is a novel oral cytotoxic agent that has demonstrated schedule-dependent clinical activity in two highly resistant cancers, malignant glioma and metastatic melanoma, as well as other refractory cancers (Stephens et al, 1987; Newlands et al, 1992; O'Reilly et al, 1993; Bleehan et al, 1995). Temozolomide, a second-generation imidazotetrazine derivative, does not require hepatic metabolism to form the cytotoxic methylating agent, 5-(3-methyltriazen-1-y1) imidazole-4-carboxamide (MTIC), whereas 5-(3,3-dimethyl-1-triazeno) imidazole-4-carboxamide (DTIC) requires hepatic activation to form MTIC (Tsang et al, 1990). Temozolomide degrades spontaneously to MTIC at physiologic pH and, therefore, is not subject to high interpatient variability in its pharmacokinetics or tissue distribution (Figure 1). Temozolomide cytotoxicity appears to be mediated principally through methylation of DNA at the $O^6$ position of guanine (Catapano et al, 1987; D'Atri et al, 1995; Wedge et al, 1996), although other mechanisms have been proposed (Liu et al, 1997).

In preclinical and clinical studies, temozolomide demonstrated extensive tissue distribution, including penetration of the blood–brain barrier and the cerebrospinal fluid (Patel et al, 1995; Data on file, Schering-Plough Research Institute; Brock et al, 1997). Temozolomide has anti-tumour activity against a variety of human tumour xenografts and murine tumour models, including glioma, melanoma, mesothelioma, sarcoma and carcinomas of the colon and ovary (Stevens et al, 1987; Plowman et al, 1994; Carter et al, 1994; Friedman et al, 1995; Wedge et al, 1997*a*). Additionally, temozolomide has demonstrated additive or synergistic anti-tumour activity when administered in vitro with other chemotherapeutic agents, radiation and inhibitors of poly (ADP-ribose) polymerase and the DNA repair protein $O^6$-alkylguanine-DNA alkyltransferase (OGAT) (Wedge et al, 1996, 1997*b*; Liu et al, 1997). OGAT is responsible for removing DNA adducts from the $O^6$ position of guanine. High levels of this protein

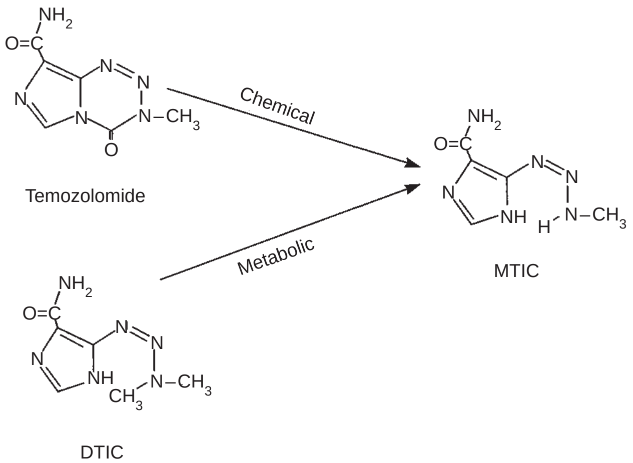

**Figure 1** Pathway for conversion of temozolomide and DTIC to the active moiety MTIC

*Received 4 January 1999*
*Revised 30 April 1999*
*Accepted 7 June 1999*

*Correspondence to:* M Brada cause resistance to temozolomide (Catapano et al, 1987; Plowman et al, 1994; Friedman et al, 1995; Waud et al, 1996; Wedge et al, 1997*a*).

Newlands et al (1992) enrolled 51 patients in a two-part phase I study to investigate the safety and efficacy of a single oral dose of temozolomide. In five patients investigated, the mean absolute oral bioavailability of temozolomide was approximately 100%. Based on the observed schedule dependency of the anti-tumour activity in mice (Stevens et al, 1987), Newlands et al (1992) administered escalating oral temozolomide once a day for 5 days to an additional 42 patients. In this population, the dose-limiting toxicity (DLT) of temozolomide was predictable and easily controlled mild to moderate myelosuppression (neutropenia and thrombocytopenia); the maximum tolerated dose (MTD) was established as 200 mg $m^{-2}$ $day^{-1}$ (Newlands et al, 1992). Consequently, a dosage of 750–1000 mg $m^{-2}$ $day^{-1}$, divided over 5 days, was recommended for phase II studies. Clinical responses were observed in patients with recurrent high-grade glioma, melanoma and mycosis fungoides (Newlands et al, 1992). Subsequent phase II studies using the 5-day schedule, repeated every 4 weeks, have confirmed clinical activity against metastatic melanoma (Bleehan et al, 1995), recurrent high-grade glioma and newly diagnosed astrocytomas (O'Reilly et al, 1993; Newlands et al, 1996; Bower et al, 1997; Levin et al, 1997). In 1993, Schering-Plough Research Institute began worldwide clinical testing of temozolomide using machine-filled capsules. This preparation of temozolomide, currently available for clinical studies, is different from the hand-filled preparation used in initial clinical studies conducted by Newlands et al (1992). The phase I trial reported here evaluates the safety, pharmacokinetics and anti-tumour activity of temozolomide, administered once daily for 5 days, repeated every 28 days, using the new machine-filled preparation. Additionally, the effect of food on the pharmacokinetics of temozolomide was assessed.

### METHODS

### Patients

All patients enrolled in this study were adults with a life expectancy of at least 12 weeks, a histologically confirmed malignancy and measurable or evaluable disease refractory to standard therapy. Additional criteria included an Eastern Cooperative Oncology Group (ECOG) performance status of > 2; white blood cell count of ≥ 4.0 × $10^9$ $l^{-1}$; platelet count of ≥ 130 × $10^9$ $l^{-1}$; haemoglobin of ≥ 10 g $dl^{-1}$; and serum creatinine and bilirubin levels within the upper limit of normal.

Patients with central nervous system (CNS) metastases, uncontrolled infection, multiple myeloma, chronic leukaemia or bone marrow involvement, as well as those who were pregnant or nursing, were excluded from the study. Also excluded were patients who had received chemotherapy, biological therapy or radiation within 4 weeks before study initiation, patients who had received nitrosourea or mitomycin C within 6 weeks before study initiation or those who experienced frequent vomiting or conditions that would prevent administration of oral capsules. Written informed consent was obtained from all patients before they entered the study.

### Study evaluations/design

Prestudy evaluations included complete blood count (white blood cells, platelets and haemoglobin), serum chemistries, chest radiograph, computerized tomography (CT) of the head, radiological assessment of the tumour (CT or magnetic resonance imaging [MRI]) and electrocardiogram (ECG). A full history was taken and a physical examination was performed on all patients before study entry and at each dosing cycle. Blood tests were performed at least once a week.

### Dose-escalation patients

To determine the nature and incidence of DLT and define the MTD, temozolomide capsules (Schering-Plough Research Institute, Kenilworth, NJ, USA) were administered orally to cohorts of three patients at an initial dosage of 100 mg $m^{-2}$ $day^{-1}$ for 5 days, followed by sequential escalation to 150, 200 or 250 mg $m^{-2}$ $day^{-1}$ for 5 days to additional three-patient cohorts, until a DLT was observed. The capsules contained 20 and 100 mg of temozolomide, and daily doses were rounded up to the nearest 20 mg to achieve 5-day dosages of 500, 750, 1000 and 1250 mg $m^{-2}$ as closely as possible. No intrasubject dose escalation was allowed. Patients were instructed to fast after midnight before each dose and to continue fasting 2 h after each dose. Patients did not receive prophylactic treatment for nausea or vomiting in the first treatment cycle.

The DLT was defined by the Common Toxicity Criteria (CTC) as grade 4 neutropenia (absolute neutrophil count of < 0.5 × $10^9$ $l^{-1}$), grade 4 anaemia (haemoglobin of < 6.5 g $dl^{-1}$), or grade 3 thrombocytopenia (platelet count of <50 × $10^9$ $l^{-1}$), serum creatinine of > 2.0 mg $dl^{-1}$, or another grade 3 or 4 adverse event (with the exception of controllable nausea or vomiting) occurring during the first course of treatment. When a DLT was encountered in one patient, a maximum of three additional patients was treated at that level. If no DLT was observed in any patients, three new patients were treated at the next-higher dosage. If a DLT was observed in any of these patients, six patients were treated at the next-lower dosage. When two patients experienced a DLT at a given dosage level, no more patients were treated at this dosage level. The MTD was defined as the dosage at which ≤ one of the six patients experienced a DLT during the first course of treatment and ≥ two patients experienced a DLT at the next-higher level. All patients continued treatment with temozolomide beyond cycle 1 until a DLT occurred or disease progressed. Grade 3–4 toxicities had to be resolved before dosing was continued.

### Food-effect patients

Fifteen patients were enrolled to assess the effect of food on the relative oral bioavailability of temozolomide in a two-way crossover design. Twelve of 15 patients met the following criteria: correct dosage of temozolomide administered on days 1 and 2 of cycle 1, pharmacokinetics evaluation performed on days 1 and 2 of cycle 1, and no occurrence of vomiting within 2 h after dosing. All patients received temozolomide, 200 mg $m^{-2}$ $day^{-1}$, once daily for 5 days, repeated every 4 weeks. Patients were randomized to one of two treatment groups: group A (fasted) or group B (fed). All patients fasted from 22:00 the previous night with water ad lib. All patients received ondansetron, 8 mg orally, 1 h before dosing as prophylaxis for nausea. On the first day of cycle 1, group A patients remained fasting for 4 h after dosing, whereas group B patients were given a modified high-fat breakfast (587 calories, 36.3 g of fat) 1 h before dosing, to be eaten within 30 min. On the second day, patients in group A followed the day 1 schedule of group B and vice versa.

*© 1999 Cancer Research Campaign*

**Table 1** Patients' characteristics

| | **All patients** ***n*** **= 30 (%)** | **Dose-escalation patients** ***n*** **= 15 (%)** | **Food-effect patients** ***n*** **= 15 (%)** |
|---|---|---|---|
| Age (years) | | | |
| Mean (range) | 50 (25–71) | 47 (25–63) | 51 (27–71) |
| Sex | | | |
| Men | 20 (67) | 11 (73) | 9 (60) |
| Women | 10 (33) | 4 (27) | 6 (40) |
| Prior therapies | | | |
| Radiation | | | |
| Yes | 19 (63) | 13 (87) | 6 (40) |
| No | 11 (37) | 2 (13) | 9 (60) |
| Surgery | | | |
| Yes | 23 (77) | 13 (87) | 10 (67) |
| No | 7 (23) | 2 (13) | 5 (33) |
| Chemotherapy | | | |
| Yes | 20 (67) | 6 (40) | 14 (93) |
| No | 10 (33) | 9 (60) | 1 (7) |
| Prior cycles of chemotherapy | 10 (33) | 9 (60) | 1 (7) |
| 0 | 6 (20) | 1 (7) | 5 (33) |
| 1 | 7 (23) | 3 (20) | 4 (27) |
| 2 | 7 (23) | 2 (13) | 5 (33) |
| ≥3 | | | |
| ECOG performance status | | | |
| 0 | 2 (7) | 1 (7) | 1 (7) |
| 1 | 20 (67) | 12 (80) | 8 (53) |
| 2 | 8 (27) | 2 (13) | 6 (40) |

Percentages may not add up to 100% as a result of rounding. Data on file, Schering-Plough Research Institute.

### Pharmacokinetics

Pharmacokinetic evaluation of temozolomide was performed during the first treatment cycle in each study. In chilled, heparinized tubes, 5-ml samples of blood were collected and cooled immediately in an ice-water bath. For the dose-escalation patients, blood samples were collected prior to dosing with temozolomide and at 10, 20 and 30 min, and then 1, 1.5, 2, 2.5, 3, 4, 6, 8, 12 and 24 h post-dose on days 1 and 5. To determine the minimum temozolomide plasma concentrations, blood samples were also collected prior to temozolomide administration on days 2, 3 and 4. For the food-effect patients, 5 ml of blood were collected prior to temozolomide administration and at 10, 20, 30, 45 min and then 1, 1.25, 1.5, 2, 2.5, 3, 4, 6, 8, 12 and 24 h post-dose on days 1 and 2.

To stabilize temozolomide in plasma and determine temozolomide plasma concentrations, plasma was separated by centrifugation at 4°C, and 2 ml was transferred to a plastic tube containing 0.1 mL of 8.5% phosphoric acid (Shen et al, 1995; Kim et al, 1997). The acidified plasma was vortexed briefly, separated into two equal portions and stored at –20°C. Plasma temozolomide concentrations were determined using a validated high-performance liquid chromatography (HPLC) assay with UV detection with a limit of quantification of 0.10 μg $ml^{-1}$ and a linear range of 0.10–20.0 μg $ml^{-1}$, using a 0.5-ml plasma sample.

Additionally, urine samples were collected on day 1 and day 5 from 0–4, 4–8 and 8–24 h after dosing to determine the amount of temozolomide excreted in the urine. Samples were collected in containers with 2 ml of 8.5% phosphoric acid. If samples had a pH of > 4, additional aliquots of 8.5% phosphoric acid were added until the pH was < 4. Samples were stored at –20°C. Urinary temozolomide concentrations were determined using a validated HPLC assay with UV detection with a limit of quantification of 1.0 μg $ml^{-1}$ and a linear range of 1.0–200 μg $ml^{-1}$ using a 0.5-ml sample. Plasma and urine assays were shown to be sensitive, specific, linear, accurate and reproducible (Shen et al, 1995).

### Pharmacokinetic parameters

Temozolomide pharmacokinetic analyses were performed using model-independent methods. The maximum plasma concentration ($C_{max}$) and time to maximum plasma concentration ($T_{max}$) were the observed values. The terminal phase rate constant (k) was calculated as the negative of the slope of the log-linear terminal portion of the plasma concentration time curve using linear regression. The elimination half-life ($t_{1/2}$) was calculated as $t_{1/2} = 0.693/k$. The area under the plasma concentration curve (AUC) from time 0 to time of final quantifiable sample (tf) was calculated using the linear trapezoidal method from start of treatment (0 h) to the last detectable plasma concentration and extrapolated to infinity as $AUC\infty = AUC_{tf} + C_{tf}/k$, where $C_{tf}$ is the estimated final concentration at tf, determined by linear regression. Since temozolomide was rapidly eliminated and did not accumulate with multiple dosing, the AUC∞ was used to approximate AUC from 0 to 24 h ($AUC_{0–24\ h}$). Total body clearance ($CL_F$) was calculated as $CL_F$ = Dose/AUC∞. The apparent volume of distribution ($V_{d/F}$) was calculated as $V_{d/F}$ = [Dose/AUC∞]/k. The accumulation ratio or index (R) was determined as the ratio of $AUC_{0–24}$ from day 5/$AUC_{0–24}$ day 1. The renal clearance ($CL_r$) was calculated as $CL_r$ = amount of temozolomide excreted in the urine from time 0 to24 h/$AUC_{0–24}$.

 *© 1999 Cancer Research Campaign*

**Table 2** Haematological toxicities: patients reporting adverse events after cycle 1

| | Dose-escalation | | | | |
|---|---|---|---|---|---|
| | ***n*** | **WHO grade** | | | |
| Dose level (mg m$^{-2}$) | | 1 | 2 | 3 | 4 |
| Thrombocytopenia | | | | | |
| 500 | 3 | 0 | 0 | 0 | 0 |
| 750 | 3 | 0 | 0 | 0 | 0 |
| 1000 | 6 | 0 | 0 | 0 | 0 |
| 1250 | 3 | 0 | 0 | 0 | 2 |
| Total | 15 | 0 | 0 | 0 | 2 (13%) |
| Anaemia | | | | | |
| 500 | 3 | 0 | 0 | 0 | 0 |
| 750 | 3 | 0 | 0 | 0 | 0 |
| 1000 | 6 | 0 | 0 | 0 | 1 |
| 1250 | 3 | 0 | 0 | 1 | 0 |
| Total | 15 | 0 | 0 | 1 (6%) | 1 (6%) |
| Neutropenia | | | | | |
| 500 | 3 | 0 | 0 | 0 | 0 |
| 750 | 3 | 0 | 0 | 0 | 0 |
| 1000 | 6 | 0 | 0 | 0 | 0 |
| 1250 | 3 | 0 | 0 | 2 | 0 |
| Total | 15 | 0 | 0 | 2 (13%) | 0 |

**Table 3** Non-haematological toxicities: number (%) of treatment-related adverse events (grades 1–4) reported for cycle 1

| Adverse events | Dose escalation | Food effects |
|---|---|---|
| Fatigue | 2 (13) | 7 (47) |
| Headache | 4 (27) | 6 (40) |
| Pain | 2 (13) | – |
| Constipation | 4 (27) | 5 (33) |
| Nausea | 12 (80) | 8 (53) |
| Vomiting | 11 (73) | 4 (27) |
| Somnolence | 5 (33) | – |
| Dizziness | – | 1 (7) |
| Fever | – | 1 (7) |
| Anorexia | – | 3 (20) |
| Diarrhoea | – | 1 (7) |

### Statistical methods

Means and standard deviations were determined for temozolomide concentration data at each time point for the dose-escalation patients. Because the sample size was small at each dose, no statistical methods were used to determine differences between doses. Individual time points and pharmacokinetic parameters (original scale AUC and $C_{max}$, $T_{max}$, k, $t_{1/2}$ $CL_F$ and volumes) were evaluated using a crossover analysis of a variance model for the food-effect evaluations. Ninety per cent confidence intervals for the mean difference between the two treatments were determined for the log-transformed AUC and $C_{max}$ values. For the food-effect evaluation, the effects of sequence of temozolomide administration, subject within sequence, phase and treatment were extracted. The pharmacokinetic parameters were analysed using the *t*-test and examined for extreme values by comparing the ranges of deviations generated from the *t*-test to the expected values derived from the analysis of variance to see if any value exceeded 3.

### Anti-tumour activity

Anti-tumour activity of temozolomide was assessed for all patients, and disease response was defined according to World Health Organization criteria. The best response for each patient was derived from the objective tumour response at each cycle. A complete response was defined as complete disappearance of all clinically detectable malignant disease, determined by two observations not less than 4 weeks apart. A partial response was defined as 50% decrease in the product of two perpendicular diameters of all lesions as determined by two observations not less than 4 weeks apart. Stable disease was defined as a < 50% decrease or a < 25% increase in the sum of the diameters of all lesions. Progressive disease was defined as a ≥ 25% increase in the size of at least one measurable lesion or the appearance of a new lesion.

### RESULTS

### Patients' characteristics

Thirty patients were enrolled in the study from 28 February, 1994, until 27 March 1995. Fifteen patients were enrolled in the dose-escalation portion of the study. Once the MTD was determined, an additional 15 patients were enrolled in the food-effect portion of the study. Patients received 120 cycles of therapy: 80 treatment cycles for the 15 dose-escalation patients and 40 treatment cycles for the 15 food-effect patients. Dose escalation was performed over 5 days as follows: three patients at 500 mg m$^{-2}$, three patients at 750 mg m$^{-2}$, six patients at 1000 mg m$^{-2}$ and three patients at 1250 mg m$^{-2}$.

The study population included patients with a range of advanced cancers. The majority had good performance status (73% ECOG ≤ 1) and, at the time of the study, had been diagnosed with cancer for at least 1 year. The most common diagnosis was glioma (10/30); the second most common was sarcoma (7/30). Most patients with glioma (8/10) had received cranial radiation, whereas only one had also undergone chemotherapy. Other tumour types, in order of incidence, included mesothelioma (3/30), ovarian carcinoma (3/30), lung carcinoma (3/30), melanoma (2/30), adenocarcinoma (1/30) and bladder carcinoma (1/30). Most patients (20/30) had received prior treatment with at least 1–2 regimens of chemotherapy (range 1–5 regimens). The majority of patients (77%) had had prior surgery. Of the 30 patients enrolled, 27 received five consecutive daily doses of temozolomide during each cycle. Patient characteristics are detailed in Table 1.

### Safety

### *Haematological toxicity*

Patients who completed at least one cycle of temozolomide or had a DLT during cycle 1 were evaluated for safety. No cumulative toxicity was observed at any dosage level when temozolomide was administered on a once-daily, 5-day schedule. Myelosuppression occurred in cycle 1 or 2 with a nadir occurring late in the cycle (day 24 to 26 for thrombocytopenia and days 30–31 for neutropenia) and rapidly recovered. Although 91% of the cycles were administered without evidence of haematological toxicity prior to dosing, five patients required a dosage reduction when haematological toxicities reappeared: three dose-escalation patients who received 1250 mg m$^{-2}$ and two food-effect patients

© 1999 Cancer Research Campaign

**Table 4** Summary of pharmacokinetic parameters from dose-escalation patients ($n$ = 15)

| | | Temozolomide | | | | | | | |
|---|---|---|---|---|---|---|---|---|---|
| | | 100 mg m$^{-2}$ day$^{-1}$ (500 mg m$^{-2}$ cycle$^{-1}$) | | 150 mg m$^{-2}$ day$^{-1}$ (750 mg m$^{-2}$ cycle$^{-1}$) | | 200 mg m$^{-2}$ day$^{-1}$ (1000 mg m$^{-2}$ cycle$^{-1}$) | | 250 mg m$^{-2}$ day$^{-1}$ (1250 mg m$^{-2}$ cycle$^{-1}$) | |
| Parameter | Unit | Mean[a] | % CV | Mean[a] | % CV | Mean[b] | % CV | Mean[a] | % CV |
| Day 1 | | | | | | | | | |
| $C_{max}$ | µg ml$^{-1}$ | 7.00 | 21 | 5.84 | 56 | 13.9 | 46 | 13.7 | 17 |
| $T_{max}$ | h | 0.50 | 0 | 0.94 | 62 | 0.94 | 87 | 1.00 | 0 |
| $AUC_{0–24\ h}$ | µg h ml$^{-1}$ | 15.5 | 8 | 17.0 | 35 | 33.2 | 15 | 43.0 | 7 |
| $AUC_{infinity}$ | µg h ml$^{-1}$ | 15.5 | 8 | 17.0 | 35 | 33.2 | 15 | 43.0 | 7 |
| $T_{1/2}$ | h | 1.72 | 4 | 1.75 | 4 | 1.79 | 6 | 1.91 | 8 |
| $Cl_{T/F}$ | ml min$^{-1}$ | 208 | 8 | 310 | 32 | 197 | 22 | 180 | 18 |
| $Cl_{T/F}$ (kg) | ml min$^{-1}$ kg$^{-1}$ | 2.48 | 10 | 4.12 | 45 | 2.54 | 17 | 2.43 | 5 |
| $V_{d/F}$ | l | 31.0 | 11 | 47.2 | 36 | 30.5 | 26 | 30.0 | 25 |
| $V_{d/F}$ (kg) | l kg$^{-1}$ | 0.37 | 9 | 0.63 | 49 | 0.39 | 13 | 0.40 | 4 |
| Day 5 | | | | | | | | | |
| $C_{max}$ | µg m$^{-1}$ | 6.92 | 30 | 5.71 | 27 | 13.0 | 39 | 12.2 | 15 |
| $T_{max}$ | h | 0.39 | 25 | 1.17 | 25 | 1.25 | 55 | 1.33 | 78 |
| $AUC_{0–24\ h}$ | µg h ml$^{-1}$ | 16.7 | 9 | 16.8 | 13 | 34.5 | 15 | 42.6 | 3 |
| $T_{1/2}$ | h | 1.81 | 4 | 1.72 | 15 | 1.79 | 9 | 1.85 | 5 |
| $Cl_{T/F}$ | ml min$^{-1}$ | 207 | 9 | 293 | 10 | 189 | 20 | 181 | 14 |
| $Cl_{T/F}$ (kg) | ml min$^{-1}$ kg$^{-1}$ | 2.48 | 13 | 3.84 | 23 | 2.45 | 18 | 2.45 | 9 |
| $V_{d/F}$ | l | 32.6 | 13 | 43.2 | 5 | 29.6 | 27 | 29.0 | 17 |
| $V_{d/F}$ (kg) | l kg$^{-1}$ | 0.39 | 15 | 0.56 | 20 | 0.38 | 16 | 0.39 | 9 |
| R | | 1.00 | 4 | 1.04 | 22 | 1.04 | 8 | 0.99 | 4 |

[a]$n$ = 3. [b]$n$ = 6. % CV = per cent coefficient of variation.

required dosage reduction to prevent haematological toxicity. The most common reason for discontinuation of treatment was progression of disease. Three dose-escalation patients discontinued the study: one patient's treatment was discontinued for an adverse event (pain in the shoulder) not attributable to the drug; one patient died at the end of the first cycle because of tumour progression; and one patient was removed from the study for administrative reasons. One patient in the food-effect portion of the study elected to discontinue treatment.

### *Dose-escalation patients*

Fifty-three of 80 (67%) cycles of therapy administered to dose-escalation patients were evaluated for haematological toxicity. Dose-limiting myelosuppression, particularly thrombocytopenia, occurred at the 1250 mg m$^{-2}$ dosage level. When temozolomide was escalated to 1250 mg m$^{-2}$, two of three patients developed CTC grade 4 thrombocytopenia, with a nadir at days 24–25 of cycle 1, and CTC grade 3 neutropenia, with a nadir on days 29 and 36. After a dosage reduction to 1000 mg m$^{-2}$, one patient remained thrombocytopenic (grade 4) and required a further reduction. Two of the three patients demonstrated grade 2 thrombocytopenia. No evidence of cumulative toxicity was reported at any dosage level in patients treated on a 5-day schedule.

### *Food-effect patients*

Grade 3 thrombocytopenia was observed after cycles 1 and 2 in 20% of patients (3/15) and was associated with grade 3 neutropenia in two patients. This was not significantly different from the incidence of grade 4 thrombocytopenia observed in the MTD cohort of the dose-escalation patients (17%). The dosage was decreased to 750 mg m$^{-2}$ in one patient with no further toxicity observed. Patients in the food-effect cohort had no grade 4 haematological toxicities.

### Non-haematological toxicity

All non-haematological toxicities were mild (CTC grade 1 or 2) and easily controlled. Table 3 presents a summary of all treatment-related non-haematological adverse events that occurred in more than one patient during the first cycle of treatment.

### *Dose-escalation patients*

The most frequent non-haematological toxicities during the first cycle for the dose-escalation patients were nausea (80%) and vomiting (73%), which occurred at all dosage levels. These toxicities usually occurred on day 1 and were considered mild to moderate in most cases. As anti-emetics were withheld until CTC Grade 3–4 nausea and vomiting occurred, this represents the actual incidence of nausea and vomiting. Gastrointestinal disturbance was generally transient, lasting an average of 1–2 days, and was relieved by ondansetron alone or in combination with metoclopramide or haloperidol. Other mild treatment-related adverse events for the first cycle included somnolence (33%), constipation (27%) and headache (27%). In all cases, the event was rated as mild to moderate. The adverse reaction profile as well as incidence and severity of adverse events remained the same in subsequent cycles.

### *Food-effect patients*

The most common treatment-related non-haematological adverse events during the first cycle of treatment for patients in the food-effect portion of the study were nausea (53%), fatigue (47%), headache (40%), constipation (33%) and vomiting (27%). The majority of adverse events, including nausea and vomiting, were mild to moderate and generally consistent with those observed for the dose-escalation patients.

*© 1999 Cancer Research Campaign*

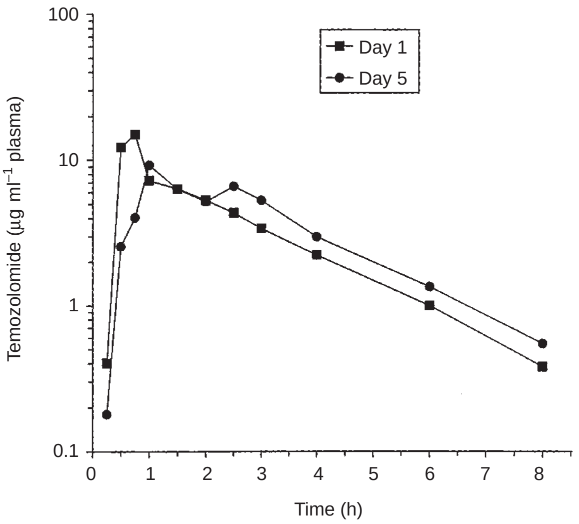

**Figure 2** Representative plasma-concentration profile for days 1 and 5 after oral administration of temozolomide 200 mg m$^{-2}$ in a patient with advanced cancer

### Pharmacokinetics

### *Dose escalation*

Single- and multiple-dose pharmacokinetics were assessed for all 15 dose-escalation patients on days 1 and 5 of cycle 1 (Table 4). Temozolomide was absorbed rapidly after oral administration of a 100–250 mg m$^{-2}$ dose of temozolomide, with a mean $C_{max}$ of 5.71–13.9 µg ml$^{-1}$ achieved within approximately 1 h (range 0.33–2.5 h) following oral administration. Elimination of temozolomide was rapid, with a mean elimination $t_{1/2}$ of 1.8 h. There was no evidence of accumulation with multiple dosing. The minimum observed plasma concentrations on days 1 through 5 were below the limit of quantification of the assay, and the mean accumulation index at each dosage level was 1.0. Dosage-related increases in $C_{max}$, $AUC_{tf}$, $AUC_{0-24}$, and AUC ∞ were observed, and the plasma concentration of temozolomide was similar on days 1 and 5 (Figure 2). The interpatient variability in the AUC observed on day 1 was small (% coefficient of variation [CV] ≤ 15) except for the 150 mg m$^{-2}$ dosage level (% CV = 35). The higher variability observed in this group was attributed to the considerably lower AUC value for one of the three patients in that group. Mean $CL_{T/F}$ values (range 2.43–4.12 ml min$^{-1}$ kg$^{-1}$) were similar on days 1 and 5 and were independent of the dosage of temozolomide. The mean $V_{d/f}$ ranged from 0.37 to 0.63 l kg$^{-1}$, suggesting that temozolomide distribution approximates that of total body water.

The recovery of unchanged temozolomide in the urine was also dosage related and ranged from 4.8% to 9.6% of the administered dose over the 24-h collection period. The mean $CL_R$ (range 0.12–0.26 ml min$^{-1}$ kg$^{-1}$) was dosage-independent and small in comparison with $CL_{T/F}$ as a result of the rapid and extensive degradation of temozolomide at physiologic pH. The mean values for urinary excretion and $CL_R$ for temozolomide are presented in Table 5.

### Effect of food

The administration of temozolomide after a modified high-fat meal had an effect on the rate and extent of temozolomide absorption (Table 6). The mean $T_{max}$ increased from 1.07 to 2.25 h ($P$ = 0.01), the $C_{max}$ decreased from 9.55 µg ml$^{-1}$ to 6.51 µg ml$^{-1}$, and the mean $AUC_{0-24}$ decreased from 30.8 to 28.1 ($P$ = 0.048) when temozolomide was administered after a meal. Although these data indicate that the presence of food results in a decrease in the rate and extent of absorption of temozolomide, the reduction in AUC was small (9.1%) and the AUC confidence levels were within 80–125% ($AUC_{0-24}$ range, 84–98%).

### Anti-tumour activity

Evidence of clinical activity was observed in 33% (10/30) of patients enrolled in the study. Stable disease was reported in nine

**Table 5** Mean urinary excretion (mg) and $CL_R$ of temozolomide on days 1 and 5 following oral administration to adult patients with advanced cancer

| | 100 mg m$^{-2}$ day$^{-1}$ | | 150 mg m$^{-2}$ day$^{-1}$ | | 200 mg m$^{-2}$ day$^{-1}$ | | 250 mg m$^{-2}$ day$^{-1}$ | |
|---|---|---|---|---|---|---|---|---|
| **Day 1** | | | | | | | | |
| **Time (h)** | **Mean ($n$ = 3)** | **% CV** | **Mean ($n$ = 3)** | **% CV** | **Mean ($n$ = 6)** | **% CV** | **Mean ($n$ = 3)** | **% CV** |
| 4 | 7.71 | 40 | 11.9 | 66 | 20.5[b] | 28 | 25.8 | 16 |
| 8 | 1.94 | 36 | 4.33[a] | 29 | 7.73 | 44 | 5.15 | 94 |
| 24 | 0.00 | – | 1.65 | 102 | 2.32 | 107 | 1.60 | 117 |
| $AUC_{0-24\ h}$ (µg h ml$^{-1}$) | 15.50 | 8 | 17.0[a] | 35 | 33.2 | 15 | 43.0 | 7 |
| $Cl_r$ (kg) (ml min$^{-1}$ kg) | 0.12 | 42 | 0.21[a] | 10 | 0.19[b] | 15 | 0.17 | 8 |
| **Day 5** | | | | | | | | |
| **Time (h)** | **Mean ($n$ = 3)** | **% CV** | **Mean ($n$ = 3)** | **% CV** | **Mean ($n$ = 6)** | **% CV** | **Mean ($n$ = 3)** | **% CV** |
| 4 | 8.26 | 24 | 15.3 | 40 | 21.3 | 48 | 29.6 | 54 |
| 8 | 1.51 | 12 | 5.01 | 45 | 7.16 | 64 | 11.4 | 55 |
| 24 | 0.36 | 173 | 0.63 | 173 | 1.61 | 96 | 3.52 | 100 |
| $AUC_{0-24\ h}$ (µg h ml$^{-1}$) | 16.7 | 9 | 16.8 | 13 | 34.5 | 15 | 42.6 | 3 |
| $CL_r$ (kg) (ml min$^{-1}$ kg) | 0.12 | 29 | 0.26 | 16 | 0.18 | 45 | 0.23 | 17 |

[a] $n$ = 2. [b] $n$ = 5.

*© 1999 Cancer Research Campaign*

**Table 6** Summary of mean pharmacokinetic parameters, point estimates and 90% confidence intervals from food-effects patients following oral administration of temozolomide (200 mg m$^{-2}$)

| Parameter | Units | Group A (fasted) $n$ = 6 | % CV | Group B (fed) $P$ | % (%)[a] | intervals[b] | Point estimate | 90% Confidence |
|---|---|---|---|---|---|---|---|---|
| $C_{max}$ | µg ml$^{-1}$ | 9.55 | 18 | 6.51 | 27 | 0.001[c] | 67.3 | 58–79 |
| $AUC_{tf}$ | µg h ml$^{-1}$ | 30.0 | 14 | 27.3 | 16 | 0.029[c] | 90.9 | 85–97 |
| $AUC_{0–24\ h}$ | µg h ml$^{-1}$ | 30.8 | 14 | 28.1 | 16 | 0.048[c] | 90.9 | 84–98 |
| $T_{max}$ | h | 1.07 | 40 | 2.25 | 48 | 0.010[d] | – | – |

[a] Expressed as a percent of treatment group A (fasted). [b]Based on log-transformed data; α = 0.05. [c]Based on log-transformed data. [d]Based on linear-scale data.

patients: four with glioma, three with sarcoma, one with mesothelioma and one with ovarian carcinoma. One patient with malignant glioma who was treated at the 1000 mg m$^{-2}$ dose level had a partial response that lasted 6.3 months. Four patients were not assessed for objective response to therapy and consequently could not be evaluated. Median time to disease progression for the four glioma patients with stable disease was 8.7 months (range 4.1–13.4 months). The median time to progression for the three sarcoma patients who experienced disease stabilization was 8.4 months (range 4.1–11.5 months).

### DISCUSSION

Newlands et al (1992) demonstrated the schedule-dependent clinical activity of temozolomide in glioma and melanoma. This study showed that temozolomide, when administered on an oral 5-day schedule as machine-filled capsules currently available for phase II clinical studies, is similar in safety and efficacy to the hand-filled capsules used in the original studies (Newlands et al, 1992).

The DLT, a rapidly reversible and noncumulative delayed thrombocytopenia, was observed at the 250 mg m$^{-2}$ day$^{-1}$ dosage level when given on a 5-day schedule, repeated every 28 days. These results are similar to the results of the first phase I study in advanced cancer, which also indicated that DLT was thrombocytopenia (Newlands et al, 1992).

The results reported here are consistent with results of a phase I study reported by the National Cancer Institute that evaluated the safety of the machine-filled temozolomide capsules in patients stratified on the basis of prior exposure to nitrosourea (Dhodapkar et al, 1997). Patients received the machine-filled capsules of temozolomide once daily for 5 days, every 28 days. The DLT for patients with and without prior exposure to nitrosourea was thrombocytopenia. The MTD for patients with prior exposure to nitrosourea was 150 mg m$^{-2}$, and the MTD for patients without prior exposure was 250 mg m$^{-2}$ (Dhodapkar et al, 1997). The MTD for temozolomide was also established as 150 mg m$^{-2}$ in a similar study that used machine-filled capsules to evaluate the safety and tolerance of temozolomide in 24 patients who were stratified by extent of prior treatment (Reidenberg, 1996). All but one of 24 patients had been pretreated with chemotherapy regimen, with or without radiation. In contrast, the majority of patients in the present dose-escalation study either had had no prior chemotherapy or had been minimally pretreated (fewer than two regimens), which may account for the slight difference observed between these studies in the dosage level that caused DLT and MTD. The results of the study reported here indicate that a 200 mg m$^{-2}$ dosage of temozolomide given on a 5-day schedule and repeated every 28 days is an appropriate dosage for future phase II studies for patients who are not pretreated with radiation and/or chemotherapy. Previous studies suggested that patients who are pretreated with chemotherapy receive a lower starting dosage of temozolomide (i.e. 150 mg m$^{-2}$), which can be escalated to 200 mg m$^{-2}$ in subsequent courses in the absence of grade 3 or 4 myelosuppression (Reidenberg, 1996; Dhodapkar et al, 1997).

The most common non-haematological side-effects associated with temozolomide were gastrointestinal toxicity with a relatively rapid onset and short duration. In all instances, it was mild to moderate and clinically manageable with standard anti-emetics. This profile is consistent with the side-effects observed in other phase I clinical studies (Newlands et al, 1992; Reidenberg, 1996; Dhodapkar et al, 1997).

The results indicated that the pharmacokinetics of temozolomide is linear and reproducible with minimal intrapatient and interpatient variability. Temozolomide was rapidly and extensively absorbed with mean peak plasma concentrations achieved within 0.33–2.5 h (mean, approximately 1 h) after oral dosing and rapidly eliminated with a mean $t_{1/2}$ of 1.8 h. The $C_{max}$ and AUC were similar after single and multiple doses, indicating that temozolomide does not accumulate in the plasma after multiple dosing. $CL_F$ was independent of dosage, indicating that temozolomide pharmacokinetics are linear. Additionally, low interpatient variability in temozolomide pharmacokinetics also reflects the fact that temozolomide does not require hepatic metabolism for conversion to MTIC. These study results indicate that the machine-filled capsule preparation of temozolomide demonstrates pharmacokinetics and clinical characteristics similar to those of the hand-filled capsules in the previous phase I study (Newlands et al, 1992), which demonstrated that temozolomide was essentially 100% bioavailable.

The results of this study indicate that emesis that occurred on day 1 did not affect the plasma concentration of temozolomide. Emesis was observed on day 1 in 11 of the 15 patients. Subsequently, all patients were given antiemetics to control nausea and vomiting post-dose on day 1 and predose on day 5. As a result, no emesis was observed on day 5. Since the day 1 and day 5 concentration versus time profiles were similar, emesis on day 1 and the administration of ondansetron predose on day 5 did not appear to affect the pharmacokinetics of temozolomide.

Administration of temozolomide after food resulted in a small decrease in its oral bioavailability. Peak plasma concentrations for the fasted patients were observed within 0.5 and 2 h (mean, 1.07 h) post-dose, whereas peak plasma concentrations for the fed patients were observed within 0.75–4 h (mean, 2.25 h) post-dose. Thus,

 *© 1999 Cancer Research Campaign*

the mean $C_{max}$ decreased by 33% when temozolomide was administered with a meal. However, the extent of absorption was only slightly reduced (9%) for the fed patients as compared with the fasted patients. The relative oral bioavailability under fed conditions ranged from 64% to 108%, with only three of the 12 patients in the fed group having a relative oral bioavailability that was ≤ 80%. Although the reduction in the extent of absorption was statistically significant ($P$ = 0.048), the decrease was small and the AUC confidence intervals were within the 80–125% guidelines for bioequivalence. Because the effect on the AUC is small, it is unlikely that the slight reduction observed in the oral bioavailability of temozolomide in the presence of a meal has any clinical significance. The incidence of emesis was similar in the fed and fasted groups, further indicating that temozolomide can be taken with food.

There was an objective response in one patient with glioma. Nine patients, including four with glioma, three with sarcoma, one with ovarian carcinoma and one with mesothelioma, had stable disease. Similar antitumor activity has been observed in phase I trials in patients with glioma, melanoma and mycosis fungoides (Newlands et al, 1992; Dhodapkar et al, 1997). Temozolomide has demonstrated clinical activity in phase II trials in metastatic melanoma (Bleehan et al, 1995) and recurrent high-grade glioma (O'Reilly et al, 1993; Newlands et al, 1996; Bower et al, 1997).

In contrast to DTIC and other alkylating agents that are prodrugs, temozolomide has the advantage of spontaneous chemical conversion to its active species, MTIC, and thus does not require hepatic metabolism. This conversion is controlled only by the pH of the local environment (Denny et al, 1994). This property will potentially result in lower patient-to-patient variability when assessing the pharmacokinetics of temozolomide. Additionally, the spontaneous conversion of temozolomide to MTIC should not be affected by the co-administration of potentially hepatotoxic agents (e.g. high-dose chemotherapy for bone marrow transplantation) and reduce the potential for interactions with other therapeutic agents.

Although temozolomide and DTIC share the same reactive moiety, MTIC, temozolomide differs from DTIC in its ability to penetrate the blood–brain barrier and cerebrospinal fluid (Patel et al, 1995; Data on file, Schering-Plough Research Institute; Brock et al, 1997). As a result, temozolomide is currently being explored for the treatment of primary and metastatic CNS tumours, particularly tumours with a high propensity for CNS metastasis, such as malignant melanoma, small-cell lung carcinoma, breast carcinoma and high-grade lymphoma.

In summary, this phase I study demonstrated an acceptable safety profile for the new chemotherapeutic agent temozolomide. The pharmacokinetics are reproducible with low interpatient variability and are only slightly modified by food. The DLT is thrombocytopenia that is reversible and non-cumulative. Temozolomide is well tolerated with manageable gastrointestinal toxicities. Unlike many other chemotherapeutic agents, temozolomide does not cause alopecia or diarrhoea, which can have a significant effect on the patient's quality of life. Temozolomide's broad spectrum of antitumour activity is encouraging and is being further confirmed in ongoing randomized phase II/III trials. Temozolomide is a well-tolerated novel oral cytotoxic agent with convenient once-daily oral administration for 5 days, a schedule that may prove to be effective in the treatment of highly resistant cancers such as recurrent glioma and metastatic melanoma.

### ACKNOWLEDGEMENTS

This study was supported by Schering-Plough Research Institute, Kenilworth, NJ, USA

### REFERENCES

Bleehen NM, Newlands ES, Lee SM, Thatcher LN, Selby P, Calvert AH, Rustin GJS, Brampton M and Stevens MFG (1995) Cancer Research Campaign phase II trial of temozolomide in metastatic melanoma. *J Clin Oncol* **13**: 910–913

Bower M, Newlands ES, Bleehen NM, Brada M, Begent RJH, Calvert H, Colquhoun I, Lewis P and Brampton MH (1997) Multicentre CRC phase II trial of temozolomide in recurrent or progressive high-grade glioma. *Cancer Chemother Pharmacol* **40**: 484–488

Brock CS, Matthews JC, Brown G, Newlands ES and Price P (1997) In vivo demonstration of $^{11}$C-temozolomide uptake by human recurrent high-grade astrocytomas [abstract]. *Br J Cancer* **75**: 1241

Catapano CV, Broggini M, Erba E, Ponti M, Marianti L, Citti L and D'Incalci M (1987) In vitro and in vivo methazolastone-induced DNA damage and repair in L1210 leukemia sensitive and resistant to chloroethylnitrosoureas. *Cancer Res* **47**: 4884

Carter CA, Waud WR and Plowman J (1994) Responses of human melanoma, ovarian, and colon tumor xenografts in nude mice to oral temozolomide. *Proc Am Assoc Cancer Res* **35**: 297 (abstract 1769)

D'Atri S, Piccioni D, Castellano A, Tuorto V, Franchi A, Lu K, Christiansen N, Frankel S, Rustum YM, Papa G, Mandelli F and Bonmassar E (1995) Chemosensitivity to triazene compounds and $O^6$-alkylguanine-DNA alkyltransferase levels: studies with blasts of leukaemic patients. *Ann Oncol* **6**: 389–393

Denny BJ, Wheelhouse RT, Stevens MFG, Tsang LLH and Slack JA (1994) NMR and molecular modeling investigation of the mechanism of activation of the antitumor drug temozolomide and its interaction with DNA. *Biochemistry* **33**: 9045–9051

Dhodapkar M, Rubin J, Reid JM, Burch PA, Pitot HC, Buckner JC, Ames MM and Suman VJ (1997) Phase I clinical trial of temozolomide (NSC 362856) in patients with advanced cancer. *Clin Cancer Res* **3**: 1093–1100

Friedman HS, Dolan ME, Pegg AE, Marcelli S, Keir S, Catino JJ, Bigner DD and Schold SC Jr (1995) Activity of temozolomide, the treatment of central nervous system tumor xenografts. *Cancer Res* **55**: 2853–2857

Kim KH, Lin CC, Parker D, Veals J, Lim J, Likhari P, Statkevich P, Marco A and Nomeir AA (1997) High-performance liquid chromatographic determination and stability of 5-(3-methyltriazen-1-yl)-imidazo-4-carboximide, the biologically active product of the antitumor agent temozolomide, in human plasma. *J Chromatogr B* **703**: 225–233

Levin V, Yung A, Prados M, Poisson M, Rosenfeld S, Brada M, Friedman H, Albright R, Olson J, Bruner J, Yue N, Dugan M and Temodal Brain Group (1997) Phase II study of Temodal® (temozolomide) at first relapse in anaplastic astrocytoma (AA) patients [abstract 1370]. *Proc Am Soc Clin Oncol* **16**: 384a.

Liu L, Chatterjee S and Gerson SL (1997) Blockade of base excision repair appears to mediate cytotoxicity to temozolomide in mismatch repair deficient tumor cells [abstract 1536]. *Proc Am Assoc Cancer Res* **38**: 288

Newlands ES, Blackledge GRP, Slack JA, Rustin GJS, Smith DB, Stuart NSA, Quarterman CP, Hoffman R, Stevens MFG, Brampton MH and Gibson AC (1992) Phase I trial of temozolomide (CCRG 81045: M&B 39831: NSC 362856). *Br J Cancer* **65**: 287–291

Newlands ES, O'Reilly SM, Glaser MG, Bower M, Evans H, Brock C, Brampton MH, Colquhoun I, Lewis P, Rice-Edwards JM, Illingworth RD and Richards PG (1996) The Charing Cross Hospital experience with temozolomide in patients with gliomas. *Eur J Cancer* **32A**: 2236–2241.

O'Reilly SM, Newlands ES, Glaser MG, Brampton M, Rice-Edwards JM, Illingworth RD, Richards PG, Kennard C, Colquohoun IR, Lewis P and Stevens MFG (1993) Temozolomide: a new oral cytotoxic chemotherapeutic agent with promising activity against primary brain tumours. *Eur J Cancer* **29A**: 940–942

Patel M, McCully C, Godwin K and Balis F (1995) Plasma and cerebrospinal fluid pharmacokinetics of temozolomide [abstract 1485]. *Proc Am Soc Clin Oncol* **14**: 461

Plowman J, Waud WR, Koutsoukos AD, Rubinstein LV, Moore TD and Grever MR (1994) Preclinical antitumor activity of temozolomide in mice: efficacy against human brain tumor xenografts and synergism with 1,3-bis(2-chloroethyl)-1 nitrosourea. *Cancer Res* **54**: 3793–3799

© 1999 Cancer Research Campaign

Reidenberg P and Villalona M (1996) Phase I clinical and pharmacokinetic study of temozolomide in advanced cancer patients stratified by extent of prior therapy [abstract 344]. *Ann Oncol* **7**: 99

Shen F, Decosterd LA, Gander M, Leyvraz S, Biollaz J and Lejeune FJ (1995) Determination of temozolmide in human plasma and urine by high-performance liquid chromatography after solid-phase extraction. *Chromatogr B* **667**: 291–300

Stevens MFG, Hickman JA, Langdon SP, Chubb D, Vickers L, Stone R, Baig G, Goddard C, Bigson NW, Slack JA, Newton C, Lunt E, Fizames C and Lavelle F (1987) Anti-tumor activity and pharmacokinetics in mice of 8-carbamoyl-3-methylimidazo (5,1-d)-1,2,3,5-tetrazin-4(3H)-one; a novel drug with potential as an alternative to dacarbazine. *Cancer Res* **47**: 5846–5852

Tsang LLH, Farmer PB, Gescher A and Slack JA (1990) Characterization of urinary metabolites of temozolomide in humans and mice and evaluation of their cytotoxicity. *Cancer Chemother Pharmacol* **26**: 429–436

Waud WR, Rubinstein LV, Kaldandrug S, Plowman J and Alley MC (1996) In vivo combination chemotherapy evaluations of topotecan with cisplatin and temozolomide [abstract 1988]. *Proc Am Assoc Cancer Res* **37**: 292

Wedge SR, Porteous JK and Newlands ES (1996) 3-Aminobenzamide and/or $O^6$ benzylguanine evaluated as an adjunct to temozolomide or BCNU treatment in cell lines of variable mismatch repair status and $O^6$-alkylguanine–DNA alkyltransferase activity. *Br J Cancer* **74**: 1030–1036

Wedge SR, Porteous JK and Newlands ES (1997*a*) Effect of single and multiple administration of an $O^6$-benzylguanine/temozolomide combination: an evaluation in a human melanoma xenograft model. *Cancer Chemother Pharmacol* **40b**: 266–272

Wedge SR, Porteous JK, Glaser MG, Marcus K and Newlands ES (1997*b*) In vitro evaluation of temozolomide combined with X-irradiation. *Anticancer Drugs* **8a**: 92–97

*© 1999 Cancer Research Campaign*
